# Acupuncture treatment on the motor area of the scalp for motor dysfunction in children with cerebral palsy: study protocol for a multicenter randomized controlled trial

**DOI:** 10.1186/s13063-019-3986-z

**Published:** 2020-01-06

**Authors:** Jun Wang, Wei Shi, Dhiaedin Khiati, Bingpei Shi, Xiaojuan Shi, Dandan Luo, Yin Wang, Rencai Deng, Huayu Huang, Jian Li, Weili Yan, Hong Yang

**Affiliations:** 1 Department of Rehabilitation, Children’s Hospital, Fudan University, Shanghai, 201102 China; 2 School of Medicine, Royal College of Surgeons in Ireland - Medical University of Bahrain, Adilya, 15503 Bahrain; 3 Clinical Trial Unit, Children’s Hospital, Fudan University, Shanghai, 201102 China; 4 Department of Rehabilitation, The 445th Hospital of Chinese People’s Liberation Army, Shanghai, 200052 China; 5 Department of Rehabilitation, Huajing Community Health Service Centre of Xuhui District, Shanghai, 200231 China; 6 Department of Rehabilitation, Jiangchuan Community Health Service Centre of Minhang District, Shanghai, 201100 China

**Keywords:** Children with cerebral palsy, Motor dysfunction, Scalp acupuncture, Study protocol

## Abstract

**Background:**

Scalp acupuncture has been widely used as treatment for motor dysfunction in children with cerebral palsy in China. Previous studies have failed to provide high-quality evidence to demonstrate the effectiveness of this treatment in children with cerebral palsy. No high-quality randomized controlled trials on scalp acupuncture have been published. The aim of this study is to evaluate the effectiveness of Jiao’s scalp acupuncture when combined with routine rehabilitation treatment versus routine rehabilitation treatment alone for motor dysfunction in children with cerebral palsy.

**Methods/Design:**

This is a four-centre randomized controlled trial. One hundred cerebral palsy patients with motor dysfunction were enrolled. Patients will be allocated in a 1:1 ratio into either an acupuncture treatment group or a control group. Cerebral palsy patients in the control group will receive conventional rehabilitation treatment, whereas patients in the acupuncture group will receive a combination of scalp acupuncture and conventional rehabilitation treatment. Thirty-six treatment sessions will be performed over a 12-week period. The Gross Motor Function Measure and the Fine Motor Function Measure Scale will be assessed as the primary outcome measures. The Paediatric Evaluation of Disability Inventory and the Cerebral Palsy Quality of Life Questionnaire for Children will be selected as secondary outcome measures. All assessments will be conducted at baseline, week 4 (treatment 12), week 8 (treatment 24), week 12 (treatment 36) and week 24 (follow-up).

**Discussion:**

This is the first trial evaluating the efficacy and safety of scalp acupuncture as a treatment for motor dysfunction in children with cerebral palsy. The results of this trial are expected to provide relevant evidence demonstrating that scalp acupuncture can be used as an effective rehabilitation treatment method for improving motor dysfunction in children with cerebral palsy.

**Trial registration:**

ClinicalTrials.gov, NCT03921281. Registered on 19 April 2019.

## Background

Cerebral palsy (CP) is a well-recognized neurodevelopmental disorder beginning in early childhood and persisting throughout the patient’s lifetime. Motor disorders are often accompanied by disturbances in sensation, cognition, communication, perception, behaviour and seizures [1–3]. CP is the most common physical disability in childhood, with a prevalence of 1.5 to 3.8 per 1000 live births in Europe, Australia and the United States [4–6]. The latest research on medical conditions, healthcare resource utilization and healthcare costs in the USA showed that standardized reimbursement costs were higher for adults with CP compared to adults without CP by a total of $16,288 in 2016 [7]. In China, 2.48 per 1000 children aged 1 to 6 years of age were affected by CP based on a survey of 12 cities. According to this estimate, approximately 5 million children with CP are among children under 14 years of age, and approximately 40,000 new cases will be diagnosed every year based on the estimated number of 16 million new-borns per year in China [8]. Due to the motor dysfunction present, children with CP show restricted activities of daily living (ADL) and social participation, which greatly influence the quality of life (QOL) and their ability to adapt to society. In addition, this places a heavy burden on families and society as a whole and becomes a significant public health issue [9].

Conventional treatment for children with CP in the West is made up of multi-professional rehabilitation, including physical therapy (PT), occupational therapy (OT) and speech therapy (ST) [10, 11]. This method is complex, and the multi-disciplinary approach is designed to minimize complications and improve the child’s function [10]. In China, acupuncture has been widely used as a treatment for children with CP in combination with the standard conventional treatment, showing promising effectiveness in improving clinical symptoms [12–16], such as drooling, sleep, bowel function, spasm, motor function and daily life activities. However, high-quality evidence to support this method as an effective treatment for children with CP is lacking [17, 18].

Many different approaches with scalp acupuncture have been used to treat motor dysfunction in children with CP in China. These include approaches such as Jiao’s, Lin’s, Jin’s, Tang’s and the China scalp-point program of the international standardization [19, 20]. However, the motor area of Jiao’s scalp acupuncture is usually the region of choice in scalp acupuncture for the treatment of motor dysfunction in CP. Jiao’s scalp acupuncture combines a modern understanding of neuroanatomy and neurophysiology with traditional techniques of Chinese acupuncture to develop a radical new tool for affecting the functions of the central nervous system and accepts a central theory that incorporates brain functions into Chinese medicine principles [10]. The motor area of Jiao’s scalp acupuncture specifically used for the treatment of motor dysfunction in CP is the equivalent of the precentral gyrus of the cerebral cortex used for scalp projection [18]. However, the exclusive effectiveness of the motor area of Jiao’s scalp acupuncture treatment on motor dysfunction in children with CP is not well documented.

Therefore, our aim is to conduct a randomized controlled trial to evaluate whether stimulation of the motor area of Jiao’s scalp acupuncture is effective in improving motor function in children with CP.

## Methods/Design

### Objectives

The objective of this proposed study is to investigate whether scalp acupuncture treatment could significantly improve motor function in children with CP.

### Study design

**Fig. 1:**
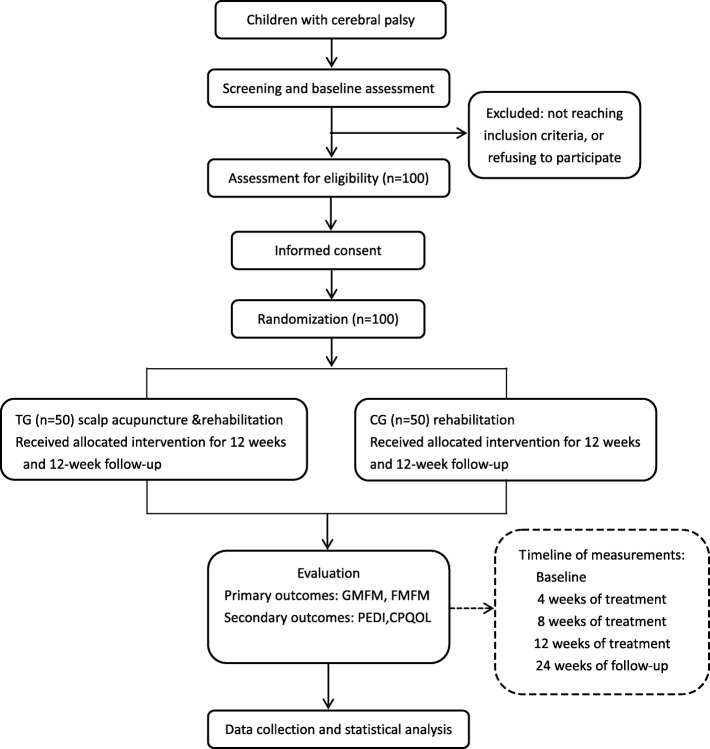
Route diagram of study design: Consolidated Standards of Reporting Trials (CONSORT) flow diagram showing subject allocation to the study conditions. *TG* treatment group, *CG* control group, *GMFM* Gross Motor Function Measure, *FMFM* Fine Motor Function Measure, *PEDI* Pediatric Evaluation of Disability Inventory, *CP-QOL* Cerebral Palsy Quality of Life

**Fig. 2:**
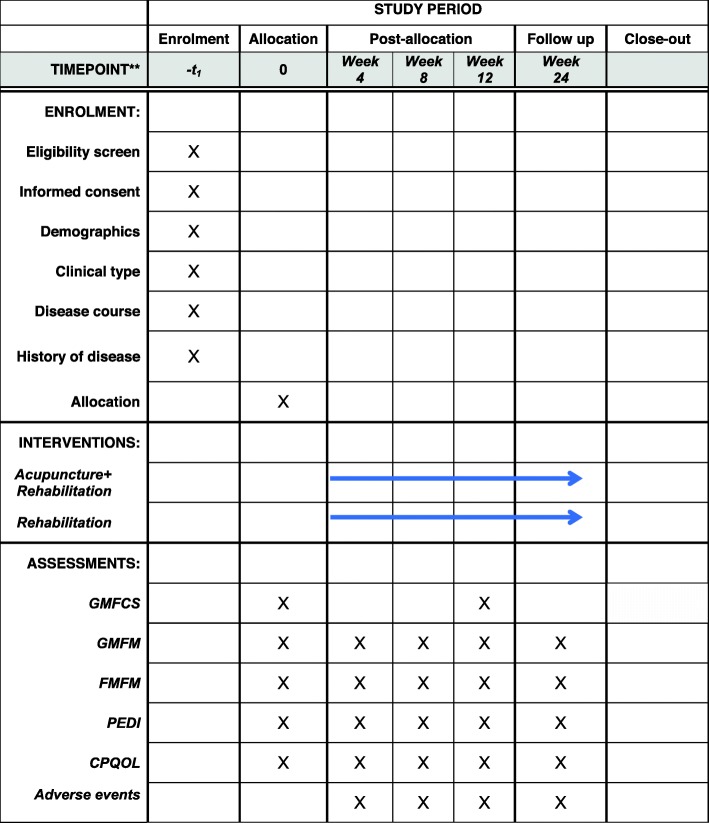
Study timeline according to the Standard Protocol Items: Recommendations for Interventional Trials (SPIRIT) diagram

This is an outcome assessor and data analyst-blinded, randomized, controlled superiority trial. The study is planned to be conducted from 1 January 2019 to 31 December 2021 in the Children’s Hospital of Fudan University. CP patients with motor dysfunction meeting the inclusion criteria will be allocated in a 1:1 ratio into either an acupuncture treatment group or a control group. CP patients in the control group will receive routine rehabilitation treatment, while the acupuncture group will receive a combination of routine rehabilitation treatment and scalp acupuncture. The Gross Motor Function Measure (GMFM) and the Fine Motor Function Measure (FMFM) will be assessed as primary outcome measures. The Paediatric Evaluation of Disability Inventory (PEDI) and the CP-Specific Quality of Life Scale (CP-QOL) will be selected as secondary outcome measurements. All assessments will be conducted at baseline, week 4 (treatment 12), week 8 (treatment 24), week 12 (treatment 36) and week 24 (follow-up). Figure 1 summarizes the flow of the entire trial. Figure 2 shows the study timeline, according to the Standard Protocol Items: Recommendations for Interventional Trials (SPIRIT) diagram. Additional file 1 presents the SPIRIT checklist.

### Inclusion criteria

Participants meeting the following inclusion criteria will be included: (1) cerebral palsy patients between 12 and 72 months old, (2) cerebral palsy was diagnosed according to the diagnostic criteria of CP found in international guidelines, (3) cerebral palsy is the spastic type, and (4) voluntary participation and informed consent signed.

### Exclusion criteria

Participants with any of the following exclusion criteria will be excluded: (1) visual, auditory and mental disorders affecting the rehabilitation assessment; (2) children with epilepsy who are uncontrolled with medication; (3) bleeding tendencies; (4) being overly sensitive to acupuncture; (5) use of muscle relaxants or herbal therapies during the study period; and (6) participation in another clinical trial.

### Informed consent

Prior to the study, the general study process will be explained by the trial coordinator to potential participants and their legal guardian. Participants and their legal guardian will be informed that participation in the trial is completely voluntary and that they can withdraw from the trial at any time. In the event of their withdrawal, study data collected on the participant will not be deleted and will be used in the final analyses. Participants will also be asked for permission for the research team to share relevant data with people from the Universities taking part in the research or from regulatory authorities, where relevant. This trial does not involve collecting biological specimens for storage. Written informed consent should be obtained from each participant and his/her legal guardian before the participant undergoes any interventions related to the study.

### Interventions

The study is a randomized clinical trial carried out in outpatient rehabilitation departments of four hospitals. A total of 100 children with CP will be recruited. The patients will be randomly assigned to two different groups: 1) the treatment group and 2) the control group. The treatment group (*n* = 50) will receive routine rehabilitation treatment combined with scalp acupuncture three times per week for 12 weeks, while the control group (n = 50) will receive routine rehabilitation treatment three times per week for 12 weeks. Both groups will be evaluated at baseline, week 4 (treatment 12), week 8 (treatment 24), week 12 (treatment 36) and week 24 (follow-up). Both groups will receive routine CP rehabilitation treatment over the course of the 12-week study period. The routine rehabilitation program was designed according to the Chinese CP rehabilitation treatment guidelines, which include physical therapy (PT) and occupational therapy (OT) for 3 days a week [21]. Chinese herbal medicine and Chinese patent drugs will be prohibited during the trial.

### Scalp acupuncture treatment

This acupuncture intervention complies with the Standards for Reporting Interventions in Clinical Trials of Acupuncture (STRICTA) guidelines. Moreover, all the acupuncturists will attend special training to achieve a sound understanding of the scalp acupuncture intervention program and to standardize the procedures performed by the different acupuncturists. The trial adheres to the STRICTA guidelines [22, 23].

**Fig. 3:**
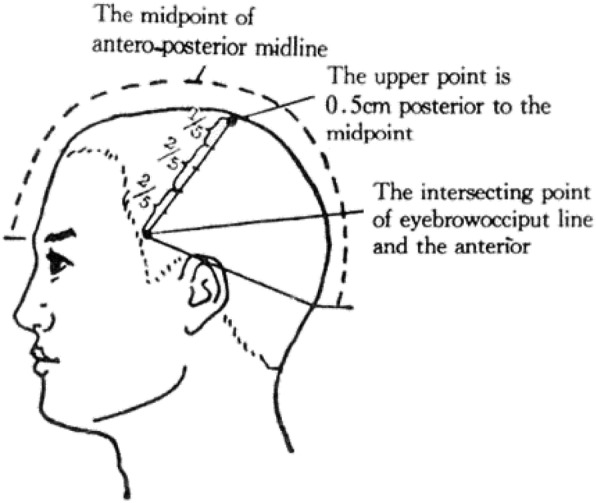
The motor area of Jiao’s scalp acupuncture

The parameters for scalp acupuncture has as the primary acupoint the motor area of Jiao’s Scalp acupuncture and as the secondary acupoint *Si shencong* (EX-HN1), which are set as follows:

1. Scalp acupoint location: The motor area of Jiao’s Scalp acupuncture is over the anterior central convolution of the cerebral cortex. It is a line starting from a point (known as the upper point of the motor area) 0.5 cm posterior to the midpoint of the anterior-posterior midline of the head and stretching diagonally to the juncture between the eyebrow-occipital line and the anterior border of the corner of the temporal hairline, which is indistinct. A vertical line extends upwards from the middle point of the zygomatic arch to the eyebrow-occipital line, and the intersection of the two lines is the projection of the motor area. The motor area is divided into five equal parts: the upper 1/5 being the motor area of the lower limbs and the trunk, the middle two-fifths being the motor area of the upper limbs and the lower two-fifths representing the motor area of the face [10] (Fig. 3 shows the motor area of Jiao’s scalp acupuncture). The site of the acupuncture treatment is determined according to the type of cerebral palsy and is based on the limb(s) affected by motor dysfunction. The motor area on the opposite side of the affected limb is selected as the site of acupuncture treatment in monoplegic and hemiplegic CP, whereas both motor areas are selected as the site of acupuncture treatment in diplegic and quadriplegic CP.

**Fig. 4:**
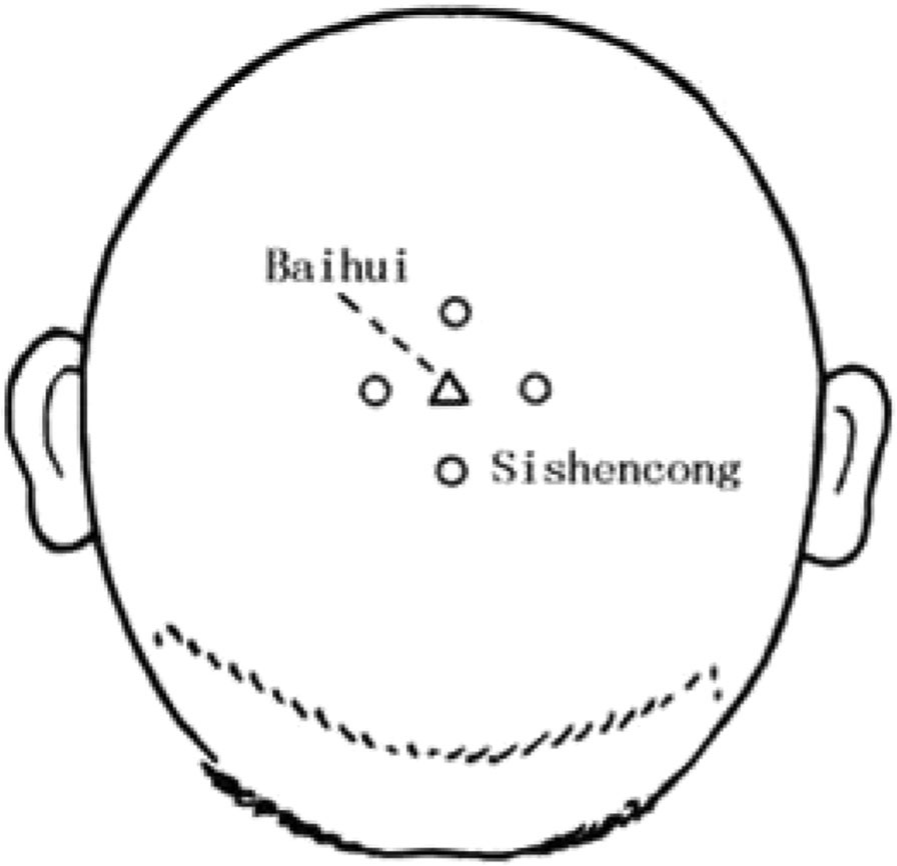
The *Si shencong* acupoints (EX-HN1)

*Si shencong* (EX-HN1) is 1 *cun* from the Baihui acupoint (Governing vessel; GV20), with one in front of it, one behind it, one on its left and one on its right, for a total of four acupoints (Fig. 4 shows the *Si shencong*). The Baihui acupoint (GV20) is 5 *cun* posterior to the front of the anterior-posterior midline of the head.

(2) Acupuncture manipulation: Disposable stainless steel needles (size 0.3 mm × 40 mm) will be manually inserted at an approximately 15-degree angle to depths of 1.5 and 2.0 cm, along the upper point and middle point of the motor area on the scalp [10]. The acupuncture direction of the *Si shencong* (EX-HN1) acupoint is toward the Baihui (GV20) acupoint. For treating motor dysfunction, the needles will be rotated for at least 200 rpm for 1 min every 20 min for a total of 60 min. Scalp acupuncture treatment will be performed by an independent certified practitioner (acupuncturist) with 5 years of clinical experience [10].
(3) Treatment course: The scalp acupuncture treatment will be implemented three times a week (once every other day), twelve times per treatment course, with each patient having three treatment courses in total.

### Rehabilitation Treatment

Children with CP will undergo the routine rehabilitation programs as mentioned above. The rehabilitation programs will be carried out three times a week (once every other day) for 12 weeks and each session of the rehabilitation treatment (PT and OT) will last approximately 1 h. All rehabilitation treatments will be carried out by qualified therapists.

### Follow-up

After the 12-week treatment observation, all patients will start an additional 12-week follow-up period. CP patients from both groups will continue to attend rehabilitation treatment in the follow-up period. However, all patients from both groups are free to choose whether to receive scalp acupuncture or not with consent from his/her parents who have given their informed consent during the follow-up period. During the 12-week follow-up period, all of the CP patients from both groups will be reassessed by GMFM, FMFM, PEDI and CP-QOL at week 24 and will be asked to fill out forms to record their rehabilitation treatment attendance. All assessment scales and forms will be returned to the researchers for review at the end of the trial.

### Outcome measures

Data collection will be performed by a trained assessor who is blind to the patients’ assignments at baseline, after the intervention (4 weeks, 8 weeks and 12 weeks) and at the end of follow-up (24 weeks).

### Basic characteristic variables

All of the participants’ general status demographic information, such as age, sex, clinical type and GMFCS level (Gross Motor Function Classification System, GMFCS) will be obtained from baseline questionnaires.

### Primary outcome measurement

The two primary outcomes of the study—Gross Motor Function Measure (GMFM) and Fine Motor Function Measure Scale (FMFM)—will be assessed at baseline, during the intervention period (at 4 weeks, 8 weeks and 12 weeks) and in the follow-up period (at 24 weeks).

### Gross Motor Function Measure

The Gross Motor Function Measure-66 (GMFM-66) is a standardized observational instrument designed to assess the gross motor function of children with CP. It is frequently utilized in clinical and research practice to measure change over time or following interventions [24]. It allows the therapist or physician to evaluate a child’s gross motor functioning by observing the way a child performs a series of motor skills. It is divided into five sections: lying and rolling; sitting; crawling and kneeling; standing; and walking, running and jumping. Each item has a very specific detailed description whereby the evaluator scores how capable the child is of completing that item on the basis of four levels: 0 = does not initiate, 1 = initiates, 2 = partially completes, and 3 = completes or NT = not tested [25]. The total score is a summation of the scores in the five areas by the Gross Motor Ability Estimator software (GMAE Version 1.0.). In addition, the Gross Motor Ability Estimator (GMAE-2) Scoring Software for the GMFM-66 can be downloaded from the CanChild website (https://www.canchild.ca/). The greater the number of tasks attempted, the greater the accuracy is of the evaluation [25].

### Fine Motor Function Measure Scale

The Fine Motor Function Measure (FMFM) assessment scale is used to evaluate the fine motor activities of children with CP, including the upper limb activities and sensory ability. This scale includes five domains, namely audio-visual tracking ability (five items), upper limb joint’s ability (nine items), grasping ability (10 items), operation ability (13 items) and hand-eye coordination (24 items), which reflect the fine motor function by a total percentage of ability. Each item has a very specific detailed description whereby the evaluator scores how capable the child is of completing that item on the basis of four levels: 0 = does not initiate, 1 = initiates, 2 = partially completes, and 3 = completes. The total score (0~100 points) is a summation of the scores in the five areas. The higher the score is, the stronger the fine motor ability [25–27].

### Secondary outcome measures

This study has two secondary outcomes—Paediatric Evaluation of Disability Inventory (PEDI) and Cerebral Palsy Quality of Life Questionnaire for Children (CPQOL)—which will be assessed at baseline, during the intervention period (at 4 weeks, 8 weeks and 12 weeks) and in the follow-up period (at 24 weeks).

### Paediatric Evaluation of Disability Inventory

Paediatric Evaluation of Disability Inventory (PEDI) is an instrument for evaluating function in children with disabilities aged 6 months to 7.5 years. The PEDI measures functional performance and capability within the three domains of (1) self-care, (2) mobility and (3) social function in three categories, that is, the Functional Skills Scale (FSS), Caregiver Assistance Scale (CAS) and Modifications Scale. The PEDI can be administered as an interview with parents/caregivers or through observation by professionals familiar with the child. The raw scores from each domain can be converted to both normative and scaled scores. The FSS covers 40 diverse content areas assessed using 197 items scored as unable (0) or capable (1). The self-care domain comprises 73 items covering the use of utensils, personal hygiene, grooming, toileting tasks and so forth. The mobility domain has 59 items covering transfers, such as normal use of toilet/potty, getting into/out of a bed or chair and indoor and outdoor locomotion. The social function domain has 65 items covering word comprehension, communication, problem-solving, playing with adults and peers and so forth [28–30]. CAS covers 20 diverse content areas scored on the following escalating six-point scale: independent, supervision, minimal help, moderate help, maximum help and total help. The items cover the self-care domain (*n* = 8), mobility domain (*n* = 7) and social function domain (*n* = 5). The Modifications Scale measures any environmental or technical modifications needed to enhance the child’s function [31].

### Cerebral Palsy Quality of Life Questionnaire for Children

The quality of life of children with CP was measured with the Chinese version of the Cerebral Palsy Quality of Life for Children (CPQOL-Child). The cerebral palsy quality of life questionnaire for children contains 66 items in seven domains: social well-being and acceptance (SWB), functioning (FUN), participation and physical health (PART), emotional well-being (EWB), access to services (ACCESS), pain and feeling about disability (PAIN) and family health (FAMILY). Almost all of the items have the following item stem: ‘How do you think your child feels about...’ and a nine-point rating scale, where 1 = very unhappy, 3 = unhappy, 5 = neither happy nor unhappy, 7 = happy, and 9 = very happy. A few items where this stem or rating scale is not appropriate, such as items in the domain of pain and feeling about disability, have the following stem and rating scale: ‘How does your child feel about the amount of pain that they have’, where 1 = not upset at all to 9 = very upset [32, 33]. The reliability and validity of the Chinese version of the CP QOL-Child have been established.

### Safety

We will conduct the following tests on all participants at the screening stage to exclude patients with serious organic lesions: white blood cell count, platelet count, haemoglobin, coagulation function, creatinine, blood urea nitrogen, alanine aminotransferase/aspartate aminotransferase, gamma-glutamyl transpeptidase and electroencephalogram examination.

The subjects will be requested to report information about adverse events (AEs). All AEs that occur during the trial period will be recorded, such as dizziness, sweating, fainting, pallor, perturbed or chest congestion during scalp acupuncture treatment, local anaphylaxis, bleeding, unbearable prickling, local hematoma, retained needle after treatment and continuous severe local pain for more than 1 hour after acupuncture. The researcher will confirm the occurrence of AEs and record all details such as the time of occurrence, date, degree, measurement related to the acupuncture treatment and causal relationship with the acupuncture treatment. Serious AEs must be reported to the principal investigator immediately [10].

### Quality control

Before the trial, all staff members are required to attend a series of training sessions. These sessions will ensure that the personnel involved fully understand the research protocol and standard operating procedures for the study. To maintain the clinical trial at a consistently high quality, the Clinical Trial Unit of Children’s hospital affiliated with Fudan university will monitor the study documents, case report forms (CRFs), informed consent forms, serious AEs and data records regularly [10].

### Adherence

First, a WeChat group will be established for the parents of the children with cerebral palsy through which they could share their child’s rehabilitation training diary once a week using their mobile phones. Secondly, a club will be established for the parents of the children with cerebral palsy, so they can share their child’s growth experience once a month at a meeting with other parents of CP patients. These strategies hopefully will be useful in improving adherence.

### Data collection, management and monitoring

The CRF, Treatment Form and Adverse Events Form will be the first to be completed and then double-entered into the electronic data capture (EDC) system by two independent investigators, who will act as the first level of control to ensure the accuracy of the data. The second level of data integrity will include data monitoring and validation, which will be performed periodically throughout the study. The original CRFs and all other forms (including the consent forms) will be archived securely in the Clinical Trail Unit (CTU) of the Children’s Hospital affiliated with Fudan University for 5 years following publication of the last paper or report from the study [10].

The safety of the study will be monitored by a data and safety monitoring board (DSMB) of the CTU of the Children’s Hospital affiliated with Fudan University, which consists of independent clinical experts and statisticians with access to unblinded data. The DSMB is independent from the sponsor, the competing interests and the investigational site and will review the performance and safety of the trial monthly [10].

The criteria for unblinding and discontinuing allocated interventions for a given trial participant include acquiring a severe disease, having serious complications of CP or experiencing serious acupuncture-related AEs (if any), which have been described previously. The DSMB will reveal a participant’s allocated intervention and make the final decision to terminate the trial [10].

The final trial dataset will be under the custody of the Children’s Hospital affiliated with Fudan University. The data manager from the CTU of Children’s Hospital affiliated with Fudan University will have access to the complete, anonymous final dataset. Access to the final dataset or identifiable data by others will require written requests to be approved by the DSMB of the CTU of Children’s Hospital affiliated with Fudan University and all study investigators [10].

### Sample size calculation

Sample size calculations were performed based on the two primary outcomes. According to our pilot trial, we assume that, after 12 weeks of treatment, the mean change in the GMFM scores in the experimental group will be greater than the control group with a mean difference of 2.4 and a standard deviation of 3.0. At an alpha level of 0.025, 40 subjects will be required for each group to ensure a statistical power of 0.9. Assuming 20% drop out, a total of 100 participants will be needed to achieve statistical significance, so each group is required to have 50 initial participants.

### Participant recruitment

Participants will be recruited in four hospitals (Children’s Hospital of Fudan University, The 445th Hospital of Chinese People’s Liberation Army, Huajing Community Health Service Centre of Xuhui District and Jiangchuan Community Health Service Centre of Minhang District) in Shanghai, China. Prospective participants will be asked to meet with the study coordinator to discuss the study and provide information about the eligibility criteria. If children with CP are eligible and their parents/guardians are interested in participating, they will be invited for a series of rehabilitation assessments after being diagnosed by neurologists. One hundred children with CP will be included in the study. When their informed consent has been obtained, children with CP will be randomized into two groups with different treatments [10].

### Randomization and allocation concealment

The recruited patients will be randomly assigned to either the experimental group or the control group according to a randomization and allocation plan. This allocation will be done using a computer-generated, block randomization (block size of 4 and 1:1 allocation) prepared by an independent epidemiologist not otherwise involved in the trial. A computer-generated block randomization process designed by the CTU is used to allocate participants to the treatment group or the control group in a 1:1 ratio (block size = 4). The randomization list is kept strictly confidential. Allocation concealment is ensured with the use of sequentially numbered (block number and sequence number), identical, opaque, sealed envelopes. Computerized randomization preserves allocation concealment and reduces the possibility of selection bias since the research assistant is kept unaware of the group assignments until after the participants are allocated to groups.

### Statistical analysis

The Statistical Product and Service Solutions (SPSS) statistical package program (version 20.0, SPSS Inc., Chicago, Ill., USA) will be used to analyse data in the CTU of Children’s Hospital affiliated with Fudan University by statisticians. All analyses, including those from any participants who drop out during the trial, will be based on the intention-to-treat (ITT) principle using the last observation carried forward rule. Missing values will be handled by the mixed model for repeated measurements. Baseline information will be collected before randomization and includes the gender and age of patients, disease course, clinical type, GMFCS level, primary outcome (GMFM, FMFM) and secondary outcomes (PEDI, CP-QOL). Descriptive statistics will be used to detail baseline participant demographics and the general status of patients, such as gender, age, disease course, clinical type and GMFCS level. Variables will be checked for normal distribution and presented as mean +/− the standard deviation and compared by Student *t* test when normally distributed. For variables not normally distributed, the data will be expressed as median +/− the interquartile range, and non-parametric tests will be used. Categorical variables will be expressed as number (%) and analysed by *χ*^2^ tests or Fisher’s exact tests, when appropriate. The mixed effect model will be used to analyse the between-group difference in repeated measures for the two primary outcomes and other outcomes (GMFM, FMFM, PEDI and CP-QOL scores) across five testing time points (weeks 0, 4, 8, 12 and 24). Mean group difference and 95% confidence intervals will be reported. Safety analyses will be compared with the incidence of AEs in the two groups using the *χ*^2^ test. A *p* value of < 0.025 will be considered as statistically significant for the two primary outcomes.

## Discussion

Chinese scalp acupuncture is a contemporary acupuncture technique integrating traditional Chinese needling methods with Western medical knowledge of the areas representative of the cerebral cortex. As acupuncture continues to develop, various physicians have begun to introduce Western neurophysiology into the field of acupuncture and explore correlations between the brain and human body. Dr. Jiao Shun-fa, who is a neurosurgeon in Shan Xi Province, is also the recognized founder of Chinese scalp acupuncture. Dr. Jiao combined the modern understanding of neurophysiology and neuroanatomy with the traditional concept of acupuncture to develop a new method of performing scalp acupuncture that can affect the functions of the central nervous system [9]. The motor area of Jiao’s scalp acupuncture is frequently used in the rehabilitation of patients paralyzed by stroke, traumatic brain injury, spinal cord injury and multiple sclerosis. It has demonstrated effectiveness in treating any type of paralysis, especially motor dysfunction after stroke [34–36]. Based on fMRI studies, scalp acupuncture has also been shown to have effects on movement regulation. Moreover, the curative effect of scalp acupuncture has been shown to be correlated with the cerebral activating reaction in motor dysfunction in stroke patients [37, 38]. The motor area of Jiao’s scalp acupuncture is divided into five equal parts: the upper one-fifth being the motor area of the lower limbs and the trunk; the middle two-fifths, the motor area of the upper limbs; and the lower two fifths, the motor area of the face [10]. However, motor dysfunction is the most significant clinical symptom in children with CP. The upper 1/5 and middle 2/5 regions of the motor area on the scalp were selected as the primary areas for scalp acupuncture stimulation while taking into account the motor dysfunction typically seen in CP. On the other hand, based on the traditional Chinese medicine theory, the disease is classified into “five kinds of retardation, five kinds of flaccidity and five kinds of stiffness”, which are the three categories of Chinese medicine according to the clinical manifestations of cerebral palsy. Furthermore, according to Chinese medicine, the disease is caused by a deficiency in the innate endowment of an individual and a deficiency the internal organs’ vital essence, which is inadequate nutrition of the “fu-viscera of mental activity”. The scalp acupoints of Si shencong (EX-HN1) are widely used in the treatment of brain-related diseases in traditional Chinese medicine, because of their effects on “activating the brain, regaining consciousness and soothing the nerves in the brain”. Therefore, the scalp acupoints of Si shencong (EX-HN1) were selected as the secondary area for scalp acupuncture stimulation [39–42]. In brief, scalp acupuncture uses special techniques to harmonize and regulate the functional activities of the brain and body.

Based on the International Classification of Functioning, Disability, and Health (ICF), once the diagnosis of CP is ascertained or highly suspected, numerous tools exist to assess the impact of CP on different health-related domains, such as physical functioning, daily activities, quality of life, health-related quality of life, family well-being, education and so on [43–46]. Cerebral palsy rehabilitation evaluation plays an important role in cerebral palsy rehabilitation clinical studies. The GMFM scale was selected as the gold standard for the evaluation of a treatment’s curative effect in almost all rehabilitation treatment CP research done both domestically and overseas. To administer the GMFM, a trained therapist observes the child completing a number of gross motor tasks in a standardised environment, and the child’s best ability is measured. The GMFM is a reliable, valid and responsive measure of gross motor function for children with cerebral palsy. It is frequently utilised in clinical and research practice to measure change over time or following interventions [24]. The FMFM assessment scale is used to evaluate the fine motor activities of children with CP, including the upper limb activities and sensory ability [47]. This scale includes five areas, namely audio-visual tracking ability, upper limb joint’s ability, grasping ability and hand-eye coordination, which reflect the fine motor function based on a score received for each ability. The PEDI was used as an individual level assessment scale to evaluate the daily life activities of CP patients, as it take into account the complex activity abilities and necessary functional skills of patients in daily environments and is often used to assess the degree of influence motor dysfunction has on families and social environments [10, 48]. The PEDI has also been recommended as a gold standard in paediatric rehabilitation [28, 29]. Quality of life is a very relevant and important construct in the context of children with cerebral palsy because it can provide a broad subjective indication of their well-being across several life domains such as physical health and social and emotional well-being. Quality of life is considered a broad and multidimensional concept that includes subjective evaluations of both the positive and negative aspects of life. In the context of rehabilitation, quality of life has clinical utility as an important health-related outcome measure that can guide practice [33, 49, 50]. Based on the concept of ICF, this study will evaluate the clinical efficacy of scalp acupuncture treatment for motor dysfunction in children with cerebral palsy in a comprehensive and multidimensional view by using the international assessment scale.

Acupuncture is a frequently used therapy for CP rehabilitation in China, but the evidence of its effect from previous studies seems to be inconclusive. Some meta-analyses have been done to study the effect of acupuncture on CP rehabilitation [17, 20]. These reviews have drawn consistent conclusions that acupuncture appears to be safe and effective for CP rehabilitation, but the benefits require further confirmation with larger, more transparent and well-conducted randomized clinical trials [10]. Thus, the purpose of this research is to observe the therapeutic effect of scalp acupuncture using Jiao’s motor area and the Si shencong (EX-HN1) acupoint for motor dysfunction in children with CP by using international general evaluation scales. Under strict quality control, this study could potentially confirm whether or not scalp acupuncture is an effective adjunct to the standard rehabilitation treatment for motor dysfunction for children with CP.

## Trial status

The treatment protocol version number currently in use is version 1.0, which was revised on 15 February 2019. Recruitment began on 1 March 2019, and the approximate date for the completion of recruitment will be 31 December 2021.

## Supplementary information

**Additional file 1.** SPIRIT checklist.

## Data Availability

The trial results will be published in a peer-reviewed scientific paper and through oral presentations at conferences. The datasets analysed during the current study are available from the corresponding author on reasonable request.

## Abbreviations

ADL: Activities of daily living
CAS: Caregiver Assistance Scale
CG: control group
CP: cerebral palsy
CPQOL: Cerebral Palsy Quality of Life Questionnaire
CRF: case report form
CTU: clinical trial unit
DSMB: data and safety monitoring board
EDC: electronic data capture
FMFM: fine motor function measure
FSS: Functional Skills Scale
GMFM: Gross Motor Function Measure
ICF: International Classification of Functioning, Disability, and Health
OT: occupational therapy
PEDI: Paediatric Evaluation of Disability Inventory
PT: physical therapy
QOL: quality of life
RCT: randomized controlled trial
ST: speech therapy
TG: treatment group
